# Supplementary material for: Exploring factors of e-waste recycling intention: The case of generation Y
Source: PLoS One. 2023 Oct 19;18(10):e0287435. doi: 10.1371/journal.pone.0287435 (PMC10586668; doi:10.1371/journal.pone.0287435)
Supplement: S1 Appendix — (DOCX) [file pone.0287435.s001.docx]

**Appendix 1**

| **Constructs** | **Items** |
| --- | --- |
| Attitude |  |
|  | E-waste recycling is pleasant. |
|  | E-waste recycling is responsible. |
|  | E-waste recycling is beneficial. |
| Subjective Norms |  |
|  | Most people who are important to me think I should engage in recycling E-waste. |
|  | Most people who are important to me would approve of me performing recycling of E-waste. |
|  | My friends/peer expects me to recycle. |
| Perceived Behavioral Control |  |
|  | I know what electronic items can be recycled. |
|  | I have full control over recycling my laptop/mobile phone/tablet. |
|  | If I wanted to, I would not have problems succeeding in recycling E-waste. |
| Normative Social Influence |  |
|  | It is important what my friends or colleagues think about E-waste recycling. |
|  | I often identify with people by recycling my E-waste recyclables. |
|  | I like to know that E-waste recycling makes a good impression on my friends or colleagues |
| Informational Social Influence |  |
|  | I often consult other people for useful information to help me choose the best alternative for e-waste recycling. |
|  | I often ask my friends for useful information to solve environmental pollution problems by recycling. |
|  | I frequently gather information from friends or colleagues about e-waste recycling. |
| Agreeableness |  |
|  | I sympathize with others’ feelings. |
|  | I am not interested in other people’s problems. |
|  | I feel others’ emotions. |
| Conscientiousness |  |
|  | I get chores done right away. |
|  | I often forget to put things back in their proper place. |
|  | Sometimes I cannot be reliable or trusted |
| Extraversion |  |
|  | It is comfortable when I am around people. |
|  | I start the conversation in most situation |
| Openness to Experience |  |
|  | Have a vivid imagination. |
|  | I am interested in abstract ideas. |
|  | I am open to new experience |
| Neuroticism |  |
|  | I have frequent mood swings. |
|  | I am relaxed most of the time |
| Recycling Intention (E-waste) |  |
|  | I am willing to contact formal e-waste recycling organizations to deal with e-waste in the future |
|  | I intend to drop off my e-waste if formal collection systems are available. |
|  | I am willing to participate in environmental programs held by the government. |
|  | I am willing to tell my relatives about the e-waste recycling experiences. |
