# Supplementary material for: Exploring factors of e-waste recycling intention: The case of generation Y
Source: PLoS One. 2023 Oct 19;18(10):e0287435. doi: 10.1371/journal.pone.0287435 (PMC10586668; doi:10.1371/journal.pone.0287435)
Supplement: Attachment — Submitted filename: Reveiwers Comments.docx [file pone.0287435.s003.docx]

**Dear Editorial Team and Reviewers,**

We appreciate the comments and suggestions made by the review and editorial team. Indeed, we think that our work has substantially improved due to these comments. We have used the track changes function in Word to highlight our modifications. In addition, our answers to the reviewers’ comments below provide additional detail and explanations as to what has been done specifically to improve the quality of the paper.

1. Reviewer Comment: This study presents a valuable contribution to understanding e-waste recycling behaviour among Lithuanian millennials. The use of the PLS-SEM approach to analyze the data is appropriate and provides robust results. The findings of the study, particularly the significant influence of attitude, subjective norms, and perceived behavioural control on consumers' e-waste recycling intention, have important implications for policymakers.
   Most of the comments were addressed well. However, the previous question of model fit is not that clear.

***Authors Response: I have mentioned the model indicators in the manuscript. I also mentioned the inner model values including saturated and estimated model values. Kindly check the data analysis part. Thanks***

2. Reviewer comment: Some papers (Determinants and mechanisms driving energy-saving behaviours of long-stay hotel guests: Comparison of leisure, business and extended-stay residential cases), (the EIAR) as well as (the JEP) had described it, the author could consider explaining more about the R square in your manuscript to help readers to clearly identify the valuable and meaningful of this value.

***Authors Response: I have incorporated further explanation of R saquare with literature support in the manuscript.***

3. Reviewer comment: Besides, it seems there is an inconsistency of the R2 value in Figure 2 (0.637) and text (0.630), please kindly check on the result.

***Authors Response: I have incorporated and corrected in the manuscript.***

4. In addition, indeed, it is some studies used the indices of R square to evaluate the model, but the value of SRMR is usually used to assess the fit of the model for the PLS-SEM method. Just a suggestion, the author can consider using second indices to discuss the fit if it is appropriate.

***Authors Response: Thanks for suggestion. Suggestion has been incorporated.***
